# Prevalence and associated factors of subclinical atherosclerosis in rheumatoid arthritis at the university hospital of Kinshasa

**DOI:** 10.1186/s41927-019-0085-4

**Published:** 2019-09-09

**Authors:** Christophe Mulumba, Pierrot Lebughe, Jean-Marie Mbuyi-Muamba, Jean-Robert Makulo, François Lepira, Jean Mukaya, Rene Westhovens, Patrick Verschueren, Jean-Jacques Malemba

**Affiliations:** 1 Rheumatology Unit, Department of internal medicine, University Hospital of Kinshasa, Kinshasa, Democratic Republic of Congo; 2 Nephrology Unit, Departement of internal medicine, University Hospital of Kinshasa, Kinshasa, Democratic Republic of Congo; 3 Radiology Unit, Department of internal medicine, University Hospital of Kinshasa, Kinshasa, Democratic Republic of Congo; 4 Department of Rheumatology, University Hospitals Leuven, Leuven, Belgium

**Keywords:** Rheumatoid arthritis, Subclinical atherosclerosis, cIMT

## Abstract

**Background:**

Rheumatoid arthritis (RA) is associated with a 5 to 10 years reduction in life expectancy due to premature atherosclerosis. This reduction is the consequence of traditional cardiovascular risk factors (TCRF) as well as systemic inflammation. The aim of the present study was to describe the prevalence and factors associated with subclinical atherosclerosis in RA at the University Hospital of Kinshasa (UHK).

**Methods:**

Patients with a diagnosis of RA based on the 2010 ACR/EULAR criteria were included in this cross-sectional study from 1 June 2014 to 31 May 2015 at the UHK. RA disease activity was measured using the DAS28-ESR. Active RA was defined by a DAS 28 > 2.6. Severe RA was defined by the presence of extra-articular manifestation, joint erosions on X-rays or HAQ ≥0.5. An assessment of subclinical atherosclerosis was performed by the measurement of the carotid intima-media thickness (cIMT) using two-dimensional ultrasonography. Subclinical atherosclerosis was defined by a cIMT ≥0.9 mm. A diagnosis of atheroma plaque was retained when the cIMT was ≥1.5 mm. The association between subclinical atherosclerosis and potential risk factors was modeled using logistic regression analysis.

**Results:**

We recruited 75 patients. The average age was 51.8 ± 14.6 years, with a sex ratio F/M of 4. The prevalence of subclinical atherosclerosis was 32%. In logistic regression being a woman of ≥55 years old (aOR 10.6, 95% CI [2.087–53.82], *p* = 0.028), DAS28-ESR > 2.6 (aOR 3.5,95% CI [1.55–10.38], *p* = 0.044), severe RA (aOR 32.6,95% CI [1.761–60.37],*p* = 0.035), high blood pressure (aOR 22.4,95% CI [5.04–99.41], *p* = 0.005) and obesity (aOR 32.3, 95% CI [2.606–40.73], *p* = 0.026) emerged as factors associated with subclinical atherosclerosis.

**Conclusion:**

Subclinical atherosclerosis is common in RA patients attending the UHK. It appears to be associated with RA disease activity and severity apart from traditional cardiovascular risk factors. These results suggest that early management of subclinical atherosclerosis targeting remaining RA disease activity and cardiovascular risk factors could slow down progression to clinical cardiovascular disease.

## Background

Rheumatoid arthritis (RA) is a chronic inflammatory rheumatic condition of autoimmune origin that affects about 0.5 to 1% of adults worldwide [1, 2]. Beyond the consequences impairing quality of life, RA is associated with a reduced life expectancy of 5 to 10 years. This is mostly due to a high incidence of cardiovascular disease (CVD) [3–6]. Cardiovascular (CV) morbidity is substantial in RA with a risk of cardiovascular events, as high as that observed in diabetic patients. This risk is 2 to 3 times higher than in the non-diabetic population [7].

This high incidence of CV morbidity and mortality results from earlier and more severe atherosclerosis compared to the general population due in part to a higher prevalence of traditional cardiovascular risk factors (smoking, diabetes, high blood pressure, dyslipidemia, ...) [8, 9]. But systemic inflammation seems to be the main cause of the high cardiovascular risk in RA. On the one hand, inflammation plays an important role in all stages of atheroma formation, from initiation to thrombosis, and on the other hand, it potentiates the effect of traditional cardiovascular risk factors [10]. The delay since diagnosis, positivity of rheumatoid factor or anti-citrullinated peptides antibodies and extra-articular manifestations also increase cardiovascular risk [11, 12].

In Democratic Republic of Congo (DRC), the relationship between atherosclerosis (ATS) and RA has not been evaluated yet. The aim of the present study was to describe the prevalence and associated factors of subclinical atherosclerosis in RA, at the University Hospital of Kinshasa (UHK).

## Methods

Consecutive RA patients were recruited between 1 June 2014 to 31 May 2015 at the rheumatology unit. Patients who suffered from another inflammatory rheumatic disease were not included in the present study. The following parameters were collected: socio-demographic data (age, sex), delay between the disease onset and the first consultation, DAS 28-ESR, joint deformities, extra-articular manifestations (rheumatoid nodes, pericarditis, pleurisies, interstitial pneumonia, etc.), cardiovascular risk factors (smoking, alcoholism, level of physical activity, hypertension, diabetes mellitus, dyslipidemia,use of glucocorticoids), blood pressure (mmHg), body weight (kg), abdominal perimeter (cm), heart rate, height (cm) and BMI (Kg / m^2^).

The diagnosis of RA was retained according to the 2010 ACR / EULAR criteria. RA disease activity was measured with the DAS28-ESR score (DAS 28 = [0. 56 x √ t28] + [0. 28 x √ sw28] + [0. 7 x Ln (ESR)] + [0.014 x (VAS-GH)]. The presence of extra-articular manifestations, joint erosions on X-ray and a HAQ ≥ 0.5 defined a severe RA. Blood pressure was measured 3 times, with measurements taken 2 min apart, using an OMRON Hem 700 IE® electronic blood pressure monitor. The patient had to be seated since at least 5 minutes and the average of the last two measurements was used for the analyses. The waist circumference (WC) was taken between the last rib and the iliac crest at the end of expiration with a tape measure. The size was taken using a SECA messband 206 cm. The weight was measured, using a KINLee DT01 scale, on a barefoot and slightly dressed patient. Pulse pressure (PP) was calculated as the difference between systolic blood pressure (SBP) and diastolic blood pressure (DBP).

The carotid intima-media (cIMT) thickness was measured by a trained sonographer. This measurement was performed at 2 cm from the carotid bulb with a LOGIQ C5 Premium color doppler ultrasound. The ultrasound system had a linear high frequency probe of 7.5 to 12 MHz. This measurement was performed on the left and right sides of the patient laying down in the supine position, arms along the body and head in moderate extension.

Upon completion of the clinical examination and ultrasonographic measurements of the cIMT, patients were referred to the UHK laboratory for blood sampling between 8 am and 9 am and after a 12 h fasting period. Fasting plasma glucose was measured using the enzymatic glucose oxidase method. Total cholesterol, triglycerides, HDL-c, uric acid were determined according to enzymatic methods with a semi-automatic device of Humalyser Primus brand. The LDL-c level was calculated using the formula of Friedewald [13]: LDL-c = CT (mg / dl) - (HDL-c) (mg / dl) -TG (mg / dl) / 5. Erythrocytes sedimentation rate (ESR) and C reactive protein were determined according to Westergren and latex agglutination methods respectively. Rheumatoid factor (RF) was measured by an ELISA method.

Hand X-rays were performed in all patients and read by a trained radiologist. Joint space narrowing, erosions and juxta-articular demineralization were looked for.

**Definitions of some concepts:**

- Early RA: duration of disease ≤2 years
- Established RA: duration of disease > 2 years
- synovitis: swollen and/or painful joints
- Obesity (WHO) [14] was defined as a BMI ≥ 30 kg / m^2^
- High blood pressure (HBP) was defined as a blood pressure ≥ 140/90 mmHg or intake of antihypertensive therapy [15].
- Diabetes mellitus was defined as two fasting glucose levels ≥126 mg/dl or, regardless of blood glucose, intake of anti-diabetic treatment [16].
- Cigarette smoking: Smoking at least 1 cigarette/day for more than 5 years or having stopped smoking for less than 5 years [17].
- Alcoholism: defined as a regular intake of 2 or more drinks of beer per day, it being understood that a glass of beer is equivalent to 10 g of alcohol [18].
- High pulse pressure (PP): PP > 60 mmHg [19].
- Tachycardia: heart rate (HR) > 90 beats / min [20].
- Physical inactivity: not being engaged in minimal physical activity defined as the equivalent of a 30 min walk three times weekly [21].
- Metabolic syndrome: was defined according to NCEP-ATP III by the presence of at least 3 of the following criteria: BP ≥ 130/85 mmHg or intake of antihypertensive treatment, waist circumference > 102 cm (man) and > 88 cm (women), HDL-c < 40 mg / dl in man and < 50 mg / dl in women, triglycerides ≥150 mg / dl), fasting blood glucose ≥100 mg / dl or antidiabetic treatment [22].
- Active RA: DAS 28 > 2.6
- Severe RA: presence of extra-articular manifestation, X-rays erosions or HAQ ≥ 0,5
- High or abnormal ESR: > 16 mm / h1 in men, and > 22 mm / h1 in women.
- Significant inflammatory syndrome: ESR ≥ 60 mm/h1 [23].
- Highly positive rheumatoid factor: RF: > 60 IU / ml [24].
- High or abnormal CRP: CRP > 6 mg / l
- Hyperuricemia: a level of uric acid> 70 mg / l in men and > 60 mg/l in women [25].
- Dyslipidemia was characterized by elevated total cholesterol ≥200 mg/dl, LDL-c ≥ 130 mg/dl, triglycerides ≥150 mg /dl, and HDL-c < 40 mg / dl in men and < 50 mg / dl in women [26].
- Subclinical atherosclerosis:cIMT ≥0.9 mm. The patient was classified as having an atheroma plaque in case of a cIMT ≥1.5 mm or in the presence of an established atheroma plaque [27].

### Statistical analysis

Data are presented as frequencies, average ± SD or medians with their extremes. Averages and median were compared using the Student t test and the nonparametric Wilcoxon/Mann Whitney tests respectively. The Pearson Chi-square or Fisher exact tests were applied to compare proportions. A multivariate regression analysis determined factors that were associated to subclinical atherosclerosis. Only variables significantly associated with subclinical atherosclerosis in univariate analysis were tested in multivariable analysis. The collinearity test was used to identify collinear variables .if two variables were collinear, only one had entered in the final model of logistic regression in multivariable analysis. Statistical analyses were performed with the SPSS software version 21.0.

## Results

75 RA patients were enrolled during the study period, including 15 males (20%) and 60 females (80%). Their average age was 51.8 ± 14.6 years. Most patients presented an active, severe and long-lasting disease. The median duration of the disease was 3 years, ranging between 2.4 months and 12 years. Symptoms of synovitis and joint deformities were observed in less than half of patients. The most common extra-articular manifestation was the presence of rheumatoid nodules (Table 1). Rheumatoid factor was negative in most patients. The biologic profile showed a dyslipidemia (elevated total cholesterol and LDLc), high median erythrocyte sedimentation rate and C reactive protein (Table 1). Radiographic lesions were present in 58.7% patients, and were dominated by juxta-articular demineralization. Erosions and joints space narrowing were less common (Table 1).

**Table 1:** Clinical, Biological and hand radiographs characteristics of RA patients in this study

Variables	*n* = 75	%
Age (years), Mean ± SD	51.8 ± 14.6	
Sex		
Men	15	20.0
Female	60	80.0
Disease activity (DAS 28-ESR)		
Mean ± SD	4.9 ± 1.5	
Remission	8	10.6
Low disease activity	5	6.7
Moderate disease activity	23	30.7
High disease activity	39	52.0
Severe RA	47	62.7
Duration of RA (years), median (extremes)	3 (0.2–12)	
Early RA	18	24.0
Established RA	57	76.0
Joint manifestations		
Synovitis	51	68
Joint deformities	26	34.7
None	15	20.0
Extra-articular manifestations		
Rheumatoid nodule	12	16
Interstitial pneumonia	2	2.7
Sicca syndrome	3	4.0
None	59	78.7
Rheumatoid factor, n (%)		
RF+	32 (42.7)	
RF-	43 (57.3)	
Glycemia, mg/dl	84.2 ± 21.7	
Uric acid, mg/dl	4.5 ± 1.6	
Total cholesterol, mg/dl	229.1 ± 48.7	
LDLc, mg/dl	161.1 ± 42.2	
HDLc, mg/dl	40.3 ± 13.5	
Triglycerides, mg/dl	147.3 ± 44.5	
ESR, mm/h1	40 (8–145)	
CRP, mg/l	12 (6–72)	
Characteristics of hand radiographs		
Normal	31	41.3
Juxta-articular demineralization	35	46.7
Erosion	4	5.3
Juxta-articular demineralization + erosion	3	4.0
Juxta-articular demineralization + erosion + joint		
space narrowing	2	2.7

*DAS 28*: Disease activity score; *SD* Standard deviation; *RF* Rheumatoid Factor; *LDLc* Low density Lipoprotein; *HDLc* High density lipoprotein; *ESR* Erythrocyte sedimentation rate; *CRP* C reactive protein

### Subclinical atherosclerosis

The average values of the cIMT were similar on the left and the right (Table 2). The prevalence of subclinical atherosclerosis in RA patients was 32%. Patients with subclinical atherosclerosis were older than those without atherosclerosis. They more often presented an active (DAS28 > 2.6) and severe (having extra-articular manifestations, erosion or HAQ ≥ 0,5) disease, as well as a higher systolic blood pressure, pulse pressure, waist circumference and BMI than those without subclinical atherosclerosis (Table 3).

**Table 2:** Mean values of cIMT in RA patients with or without subclinical atherosclerosis

Variables	all n = 75	ATS – *n* = 51	ATS+ *n* = 24
cIMT, (mm)			
cIMT right	0.74 ± 0.17	0.64 ± 0.11	0.95 ± 0.07
cIMTleft	0.73 ± 0.18	0.62 ± 0.11	0.95 ± 0.06

*cIMT* Carotid intima-media thickness

**Table 3:** Clinical, biological characteristics and traditional cardiovascular risk factors of patients with or without subclinical atherosclerosis

Variables	ATS+ (n = 24)	ATS-(n = 51)	p
Age (years)	60.4 ± 11.0	47.8 ± 14.4	< 0.0001
Female age ≥ 55 years,n (%)	15 (75.0)	16 (40.0)	0.010
Male age ≥ 45 years,n (%)	4 (100.0)	10 (55.6)	0.137
Established RA, n (%)	15 (62.5)	42 (82.4)	0.005
Median duration of RA (years)	2 (0.3–12.0)	2 (0.2–12.0)	0.850
DAS28-ESR > 2,6 n (%)	23 (95.8)	39 (76.5)	>0.034
Severe RA, n (%)	18 (66.7)	29 (56.9)	0.029
Dose of MTX, mg/week	7.8 ± 1.5	8.5 ± 2.2	0.153
Duration of corticosteroid use, month, median (extremes)	1 (1.0–6.0)	2 (0.5–12.0)	0.975
Heart rate, bpm	81.1 ± 15.2	81.3 ± 10.2	0.949
SBP, mmHg	137.9 ± 19.4	125.3 ± 19.5	0.010
DBP, mmHg	79.1 ± 9.4	77.2 ± 10.8	0.447
PP, mmHg	58.3 ± 18.7	49.9 ± 14.2	0.034
Waist cicumference, cm	94.9 ± 11.3	86.7 ± 11.8	0.006
BMI, Kg/m2	26.7 ± 4.9	22.7 ± 4.2	0.001
HBP, n (%)	14 (58.3)	7 (13.7)	< 0.0001
Diabetes mellitus, n (%)	7 (29.2)	3 (5.9)	0.010
Physical inactivity, n (%)	19 (79.2)	22 (43.1)	0.003
Alcoholism, n (%)	8 (33.3)	13 (25.5)	0.330
Tobacco use, n (%)	1 (4.2)	1 (2.0)	0.541
Global Obesity, n (%)	8 (33.3)	1 (2.0)	< 0.0001
Overweight, n (%)	9 (37.5)	13 (25.5)	0.212
PP > 60 mmHg, n (%)	7 (29.2)	15 (29.4)	0.603
Metabolic syndrome, n (%)	9 (37.5)	10 (19.6)	0.008
Low HDL-c, n (%)	7 (29.2)	13 (25.5)	0.471
Hypertriglyceridemia, n (%)	16 (66.7)	20 (39.2)	0.024
Hypercholesterolemia, n (%)	19 (79.2)	31 (60.8)	0.009
High LDL-c,n (%)	18 (75.0)	35 (68.6)	0.390
Glycaemia,mg/dl	85.1 ± 22.4	83.7 ± 21.6	0.798
RF+, n (%)	8 (33.3)	24 (47.1)	0.192
ESR,mm/h1,median (extremes)	40.5 (8–115)	40.0 (13–145)	0.866
CRP, median (extremes)	24.0 (6–72)	21 (6–72)	0.775

*RA* Rheumatoid arthritis; *MTX* Methotrexate; *HR* Heart rate; *bpm* Beats per minute; *PP* Pulse pressure;*SBP* Systolic blood pressure; *DBP* Diastolic blood pressure; *BMI* Body mass index; *RF* Rheumatoid factor; *ESR* Erythrocyte sedimentation rate; *CRP* C reactive protein. *HBP* High blood pressure; *HDLc* High density lipoprotein; *LDLc* Low density lipoprotein; *SD* Standard deviation

The prevalence of traditional cardiovascular risk factors was higher in patients with subclinical atherosclerosis than other patients (Table 3). These factors were: being a female ≥55 years old, hypertension, diabetes mellitus, physical inactivity, obesity, metabolic syndrome, hypertriglyceridemia, hypercholesterolemia and hyperuricemia.

In univariate analysis age ≥ 55 years in females, active RA (DAS28-ESR > 2,6), severe RA, hypertension, diabetes mellitus, physical inactivity, obesity, hypertriglyceridemia and metabolic syndrome were the main factors associated with subclinical atherosclerosis (Table 4). In multivariable analysis this association persisted only for age ≥ 55 years in female, active disease, severe disease, hypertension and obesity. Risk increased 11-fold in women ≥55 years old, 33-fold in patients with severe disease, 22-fold in hypertensive, and 32-fold in obese patients (Table 4)..

**Table 4:** Multivariate regression analysis of factors associated with subclinical atherosclerosis

Variables	Univariateanalysis		Multivariateanalysis	
	OR (95%CI)	p	aOR (95%CI)	p
Age (women)				
< 55 years	1		1	
≥ 55 years	4.5 (1.36–14.84)	0.014	10.6 (2.09–53.82)	0.028
Active RA				
Inactive	1		1	
DAS 28-ESR>2.6	7.5 (1.85–9.16)	0.007	3.5 (1.16–10.38)	0.044
Severe RA				
Not severe	1		1	
severe	3.6 (1.13–4.74)	0.007	32.6 (1.76–60.37)	0.035
High sedimentation rate				
No	1		1	
yes	5.0 (1.53–7.29)	0.016	1.0 (0.99–1.08)	0.123
HBP				
No	1		1	
yes	15.0 (2.18–20.04)	0.006	22.4 (5.04–29.41)	0.005
Diabetes mellitus				
No	1		1	
yes	7.0 (1.11–14.06)	0.038	1.5 (0.31–3.09)	0.216
Physical inactivity				
No	1		1	
yes	8.3 (1.88–12.01)	0.006	1.4 (0.48–4.81)	0.189
Global obesity				
No	1		1	
yes	25.0 (2.9–45.42)	0.003	32.3 (2.61–40.73)	0.026

*RA* Rheumatoid arthritis; *SR* Sedimentation rate; *HBP* High blood pressure; *aOR* Adjusted odds ratio

## Discussion

The present study described the prevalence of subclinical atherosclerosis in Congolese RA patients and identified its determinants.

The average age of participants was 51.8 ± 14.6 years. This is similar to the mean age reported in previous studies conducted in Kinshasa [2, 28, 29]. It should be reminded that RA occurs most often between 35 and 50 years of age. The female predominance observed in the present study confirms what is described in the literature [30]. More than half of patients had an established and active RA with a median disease duration of 3 years. This observation suggests that patients consulted rather late. This delay could be the result of ignorance or under-reporting of patients, poverty, the use of traditional medicine and especially self-medication.

The proportion of patients with rheumatoid factor was lower than those reported in Senegal [31], Togo [32] and especially in the Western countries [33]. A similar observation has already been made in previous studies conducted in DR Congo [28]. The fact that more than half of the patients were rheumatoid factor negative potentially contributes to the less destructive nature of the disease, as evidenced by the rarity of articular erosions on radiography [34].

Three out of 10 patients had subclinical atherosclerosis. This frequency is similar to those reported by Kassem et al. in 2010 (33.3%) [25], Hannawi et al. (2007) [35%], Grovers et al. [33.3%] [35] and Gonzalez et al. in 2003 (34%) [26]. On the other hand, it was higher than that reported by Mahajan et al. in 2008 (21%) [36] and lower than that reported by Mohan et al. in 2014 (59.3%) [37]. Differences in the frequency of subclinical atherosclerosis between studies may, in part, be explained by the difference in the thresholds used to define subclinical atherosclerosis.

Patients with subclinical atherosclerosis had a significantly higher SBP and PP values than those without atherosclerosis. Indeed, the association between SBP, PP and subclinical atherosclerosis generally reflects the loss of the elasticity of the arterial wall of the aorta which becomes rigid, explaining the elevation of SBP as well as the decrease of DBP; and consequently the elevation of the PP [38].

The present study did not show a significant association between subclinical atherosclerosis and the duration of the disease. These results corroborate those of Jonsson et al. [39], as well as Alkaabi et al. [40]. In contrast, Kumeda et al. and Park et al. reported an association between disease duration and subclinical atherosclerosis. So, chronic inflammation is the basis of long-term progression of RA which is closely linked to the development of subclinical atherosclerosis [41, 42].

Being a woman ≥55 years old, disease activity level and severity of the disease, hypertension as well as obesity emerged as the main factors independently associated with subclinical atherosclerosis. The impact of age in the development and progression of atherosclerosis has been demonstrated in several studies [43, 44]. Age is a potent cardiovascular risk factor. It participates in the appearance of vascular lesions induced by the oxidative stress and inflammation. These lesions lead to the development of other risk factors such as hypertension, and atherosclerosis. In addition to that, age in women ≥55 years corresponds to the menopausal period which contributes to the occurrence of subclinical atherosclerosis. Indeed, the decrease in oestradiol during menopause results in an atherogenic lipid profile and endothelial dysfunction responsible for an increased cardiovascular risk [44]. Our results are similar to those of Dionicio et al. reported in 2012 [45]. In contrast, Rawhya et al. reported only an association with disease activity [46].

The present study did not observe an association between CRP and subclinical atherosclerosis. This result must be considered with caution because of the small size of our sample. Apart from that, we have used a quantitative method of CRP with a cutoff of 6 mg/l (the most commonly available in our country). With this method CRP values and cutoffs are higher compared to the ultrasensitive method and may have underestimated the number of patients with high CRP level. So, it is possible that by using an ultrasensitive method, we might have found an association between CRP and subclinical atherosclerosis.

The activity and severity of the disease have also been implicated in the occurrence of subclinical atherosclerosis. Systemic inflammation, which can precede the onset of joint symptoms in RA, may favor the development of atheromatous disease before joint manifestations. The pathogenesis of atherosclerosis is related to that of autoimmune joint disease [47, 48, 49]. Systemic inflammation may also be associated with obesity (pro-inflammatory state) and induce insulin resistance, changes in lipid profile and, consequently, development of metabolic syndrome in patients with active RA [50, 51]. So, the control of inflammation and management of traditional risk factors should normally reduce the occurrence of early atherosclerosis.

The present study has some limitations. First, the small sample size cannot provide enough power for the statistical tests used to detect possible associations. Second, the cross-sectional nature of this study excludes any possibility to establish a causal relationship. Third, the lack of measurement of ACPA in our study is also a limitation since ACPA may be related to the increasing of cardiovascular risk. These limitations may be solved by a case-control study or a longitudinal study on a large sample.

However, this study has the merit of being the first to describe the prevalence of subclinical atherosclerosis in Congolese RA patients and to identify its associated factors. Moreover, it shows that the measurement of the cIMT may be used as a simple and reliable tool for detecting subclinical atherosclerosis, and identifying patients who are most likely to benefit from preventive measures.

## Conclusion

The current study showed that subclinical atherosclerosis is frequent (32%) among patients suffering from rheumatoid arthritis at the University Hospital of Kinshasa. It is associated with traditional cardiovascular risk factors and RA disease characteristics. Patients with subclinical atherosclerosis had a more active and severe disease than those without subclinical atherosclerosis. These results suggest that screening and early management of subclinical atherosclerosis and risk factors in patients with RA could slow down the progression to clinical cardiovascular disease.

## Data Availability

All Data used and analysed during the current study are available from the corresponding author on reasonable request.

## Abbreviations

ACR: American College of Rheumatology
aOR: Adjusted odds ratio
cIMT: Carotid intima-media thickness
CRP: C reactive protein
CV: Cardiovascular
CVD: Cardiovascular disease
DAS-28: Disease activity score
DBP: Diastolic blood pressure
ESR: Erythrocyte sedimentation rate
EULAR: European League against Rheumatism
HAQ: Health assessment questionnaire
HDLc: High density lipoprotein
LDLc: Low density lipoprotein
PP: Pulse pressure
RA: Rheumatoid arthritis
RF: Rheumatoid factor
SBP: Systolic blood pressure
SD: Standard deviation
TCRF: Traditional cardiovascular risk factors
UHK: university hospital of Kinshasa
